# Prevalence of hearing loss, tinnitus, vertigo and sudden deafness among patients with polymyositis and dermatomyositis

**DOI:** 10.1038/s41598-024-56372-z

**Published:** 2024-03-09

**Authors:** Shih-Han Hung, Sudha Xirasagar, Luong Huu Dang, Nguyen-Kieu Viet-Nhi, Yen-Fu Cheng, Chin-Shyan Chen, Herng-Ching Lin

**Affiliations:** 1 Department of Otolaryngology, School of Medicine, Taipei Medical University, Taipei, 110 Taiwan; 2 Department of Otolaryngology, Wan Fang Hospital, Taipei, 110 Taiwan; 3 International Ph.D. Program in Medicine, College of Medicine, Taipei Medical University, Taipei, 110 Taiwan; 4 Department of Health Services Policy and Management, Arnold School of Public Health, University of South Carolina, Columbia, SC USA; 5 Department of Otolaryngology, Faculty of Medicine, University of Medicine and Pharmacy at Ho Chi Minh City, Ho Chi Minh City, Vietnam; 6 Department of Medical Research, Taipei Veterans General Hospital, Taipei, Taiwan; 7 Department of Otolaryngology-Head and Neck Surgery, Taipei Veterans General Hospital, Taipei, Taiwan; 8 Department of Otolaryngology-Head and Neck Surgery, School of Medicine, National Yang Ming Chiao Tung University, Taipei, Taiwan; 9 Research Center of Data Science on Healthcare Industry, College of Management, Taipei Medical University, Taipei, Taiwan; 10 Department of Economics, National Taipei University, New Taipei City, Taiwan; 11 School of Health Care Administration, College of Management, Taipei Medical University, Taipei, Taiwan; 12 Research Center of Sleep Medicine, Taipei Medical University Hospital, Taipei, Taiwan

**Keywords:** Tinnitus, Polymyositis, Dermatomyositis, Inner ear disorders, Epidemiology, Immunology, Neurology, Rheumatology

## Abstract

Little is known about a possible association of autoimmune inner ear disease among patients diagnosed with polymyositis (PM)/dermatomyositis (DM). This study aimed to explore differences in the prevalence of inner ear symptoms among patients with and without PM/DM using a nationwide population-based dataset. Data for this study were retrieved from the Taiwan National Health Insurance Research Database. The study sample included 1622 patients diagnosed with PM/DM and 8109 propensity-score matched comparison patients without PM/DM. We performed multivariate logistic regressions to calculate odds ratios (ORs) and 95% confidence interval (CI) for tinnitus, hearing loss, sudden deafness, and vertigo among patients with PM/DM versus comparison patients. Chi-square tests showed statistically significant differences between patients with PM/DM and comparison patients in the prevalence of tinnitus (16.1% vs. 12.7%, *p* < 0.001), non-conductive hearing loss (9.2% vs. 6.8%, *p* < 0.001), and vertigo (14.4% vs. 11.1%, *p* < 0.001). The adjusted ORs for tinnitus, non-conductive hearing loss, and vertigo, respectively, were 1.332 (95% CI = 1.147–1.547), 1.399 (95% CI = 1.154–1.696), and 1.374 (95% CI = 1.173–1.611) for patients with PM/DM when compared to comparison patients. Our study finds that patients with PM/DM have higher prevalence rates of tinnitus, non-conductive hearing loss, and vertigo than comparison patients.

## Introduction

Immunologically-mediated disease of the inner ear could be due to direct effects of the immune response or indirect effects of deposits of circulating immune complexes caused by systemic immune-mediated diseases in the inner ear^1–4^. Although autoimmune inner ear disease (AIED) is rare, accounting for less than 1% of all cases of hearing impairment or dizziness, autoimmune etiology is often overlooked owing to the absence of a specific diagnostic test^4–6^. Studies report vertigo, hearing loss, and aural fullness among 50–80% of patients diagnosed with AIED, making it challenging to distinguish AIED from Meniere's disease^5–8^. When the inner ear is the only organ affected by the autoimmune process, the AIED is considered “primary”, and in 15–30% of AIED, it is “secondary” due to occurring in the context of systemic autoimmune disease^3^. Several studies have shown a relationship between sensorineural hearing loss (SNHL) and systemic autoimmune diseases (SDA), especially correlations between hearing-vestibular clinical symptoms and pathological mechanisms involved in systemic lupus erythematosus, rheumatoid arthritis, polyarteritis nodosa, and Cogan's syndrome^4,8–12^.

Polymyositis (PM) and Dermatomyositis (DM) are rare autoimmune inflammatory myopathies, often associated with cancer and significant premature mortality^13,14^. Little is known about possible associations between AIED and PM/DM. Acknowledging such a correlation would enable prompt detection of prospective auditory issues amongst individuals with PM/DM, thereby enhancing their quality of life^3,15^.

This study aims to explore co-occurrence of inner ear disorders and PM/DM with either condition preceding the other, using a retrospective observational study of population-based data from Taiwan’s Longitudinal Health Insurance Database.

## Methods

### Study design and data source

This retrospective observational study used data from the Longitudinal Health Insurance Database 2010 (LHID2010), which is derived from the Taiwan National Health Insurance (NHI) program. Taiwan launched its single-payer, universal coverage, health insurance system in 1995. The LHID2010 comprises registration files and original medical claims for 2,000,000 enrollees randomly selected from all NHI enrollees listed in the 2005 Registry of Beneficiaries, representing Taiwan’s population of 23.72 million citizens). The Taiwan National Health Research Institutes and other researchers have validated the completeness, accuracy, and population representativeness of the LHID2010. In addition, many researchers from Taiwan have used the LHID2010 to publish epidemiological studies in international peer-reviewed journals. The LHID2010 provides an excellent opportunity to explore otologic manifestations in patients with PM/DM.

The study was approved by the institutional review board of Taipei Medical University (TMU-JIRB N202211048) and is compliant with the Declaration of Helsinki. Because we used deidentified administrative data, the Institutional Review Board of Taipei Medical University waived the need for patient informed consent requirement.

### Identification of study patients

We designed this cross-sectional study by comparing the study group (patients with a diagnosis of DM or PM) with a comparison group. For the study group, we first extracted the claims of all patients aged over 20 years showing a diagnosis of DM (ICD-9-CM code 710.3 or ICD-10-CM M33. 1) or PM (ICD-9 710.4 or ICD-10 M33.2) in ambulatory care visits to clinics or outpatient departments of hospitals between January 1, 2015, and December 31, 2018. We included patients with at least two separate visits during the study period showing the diagnosis in order to improve diagnostic validity. As a result, 1622 patients with PM/DM were identified during the study group.

We selected a comparison group from the remaining enrollees in the LHID2010 aged ≥ 20 years. Of them all enrollees who had ever received a PM/DM diagnosis or other autoimmune diseases prior to January 1, 2015 were excluded. Thereafter, propensity score matching was used to select comparison patients. We first calculated a propensity score for each enrollee using demographic variables likely to impact the occurrence of PM/DM, age, sex, monthly income (NT)$0–15,840, NT$15,841–25,000, ≥ NT$25,001; the average exchange rate in 2022 was US$1 ≈ NT$29), geographic location (Northern, Central, Southern, and Eastern), and urbanization level of the patient’s residence (5 levels, 1 most urbanized, 5 least urbanized) and medical comorbidities (hypertension, coronary heart disease and diabetes). We used matching ratio of five comparison patients to one patient with PM/DM, for a final study sample of 1622 patients with PM/DM and 8109 matched patients without PM/DM (nearest neighbor method).

### Measures of outcomes

The selected outcome variables were disorders of the inner ear, tinnitus, hearing loss, sudden deafness, and vertigo occurring either after or before the diagnosis of PM/DM during the study period. Cases were identified using ICD diagnosis codes as follows: tinnitus (ICD-9-CM code 388.3 or ICD-10-CM codes H93.1, H93.11, H93.12, H93.13, H93.19), non-conductive hearing loss (ICD-9-CM code 389 excluding 389.0 or ICD-10-CM codes H90 excluding H90.0–H90.2, H91), sudden deafness (ICD-9-CM code 388.2 or ICD-10-CM codes H91.2) or vertigo (ICD-9-CM code 386.1, 386.10, 386.19 or ICD-10-CM codes H81.391, H81.311, H81.312, H81.313).

### Statistical analysis

Statistical analyses were carried out using the SAS system (SAS System for Windows, vers. 9.4, SAS Institute, Cary, NC). Chi-square test and t-tests were used to study differences between the study group and comparison group on demographic characteristics and the occurrence of tinnitus, non-conductive hearing loss, sudden deafness and vertigo. We used multivariable logistic regression to calculate the odds ratios (OR, 95% confidence interval (CI)) for tinnitus, non-conductive hearing loss, sudden deafness, and vertigo in the PM/DM group versus comparison group. Two-sided *p* of 0.05 was used to determine statistical significance.

### Ethical approval

The study was approved by the institutional review board of Taipei Medical University (TMU-JIRB N202211048).

## Results

Table 1 shows that the study group and comparison group were similar on sociodemographic characteristics, age (*p* = 0.841), sex (*p* = 0.996), monthly income (*p* = 0.989), geographic location (*p* = 0.931), and residential urbanization level (*p* = 0.998). In addition, there were no significant associations in hypertension (*p* = 0.996), coronary heart disease (*p* = 0.998) and diabetes (*p* = 0.995) between study group and comparison group.

**Table 1:** Demographic characteristics of persons with polymyositis or dermatomyositis and comparison patients (n = 9731).

Variables	Patients with polymyositis/dermatomyositis (*n* = 1622)		Propensity score-matched comparison patients (*n* = 8109)		*P* value
	Total no	%	Total no	%	
Age, mean (SD)	50.6 (15.7)		50.7 (15.8)		0.841
Males	674	41.6	3369	41.6	0.996
Monthly income					0.989
< NT$1–15,841	342	21.1	1699	21.0	
NT$15,841–25,000	660	40.7	3314	40.9	
≥ NT$25,001	620	38.2	3096	38.2	
Geographic region					0.931
Northern	626	38.6	3151	38.9	
Central	455	28.0	2269	28.0	
Southern	509	31.4	2548	31.4	
Eastern	32	2.0	141	1.7	
Urbanization level					0.998
1 (most urbanized)	470	29.0	2340	28.9	
2	488	30.1	2415	29.8	
3	290	17.9	1461	18.0	
4	220	13.6	1109	13.7	
5 (least urbanized)	154	9.5	784	9.7	
Hypertension	842	51.9	4210	51.9	0.996
Coronary heart disease	463	28.6	2315	28.6	0.998
Diabetes	491	30.3	2454	30.3	0.995

Table 2 presents the prevalence rates of tinnitus, non-conductive hearing loss, sudden deafness, and vertigo among the total study sample, and the study- and comparison groups. Among the total sample, prevalence rates of tinnitus, non-conductive hearing loss, sudden deafness, and vertigo were 13.3%, 7.2%, 1.1%, and 11.7%, respectively. Chi-square tests showed statistically significant differences between patients with PM/DM and comparison patients in the rates of tinnitus (16.1% vs. 12.7%, *p* < 0.001), non-conductive hearing loss (9.2% vs. 6.8%, *p* < 0.001), and vertigo (14.4% vs. 11.1%, *p* < 0.001). However, there was no difference in the rates of sudden hearing loss between the two groups (1.0% vs. 1.1%, *p* = 0.662).

**Table 2:** Prevalence rates of tinnitus, non-conductive hearing loss, sudden deafness and vertigo among patients with polymyositis/dermatomyositis vs. comparison patients.

Variable	Total (*n* = 9731)	Patients with polymyositis/dermatomyositis (*n* = 1622)	Comparison patients (*n* = 8109)	*P* value
	*n*, %			
Presence of tinnitus	1289 (13.3)	261 (16.1)	1028 (12.7)	< 0.001
Presence of non-conductive hearing loss	703 (7.2)	149 (9.2)	554 (6.8)	< 0.001
Presence of vertigo	1134 (11.7)	234 (14.4)	900 (11.1)	< 0.001
Presence of sudden deafness	106 (1.1)	16 (1.0)	90 (1.1)	0.662

Table 3 presents the results of logistic regression analyses, showing ORs for tinnitus, non-conductive hearing loss, and vertigo of 1.321 (95% CI = 1.140–1.531), 1.380 (95% CI = 1.142–1.668), and 1.351 (95% CI = 1.157–1.577), respectively, for patients with PM/DM relative to the comparison group. The adjusted analysis closely mirrored the single-variable analysis, with adjusted ORs of 1.332 (95% CI = 1.147–1.547), 1.399 (95% CI = 1.154–1.696), and 1.374 (95% CI = 1.173–1.611) for tinnitus, non-conductive hearing loss, and vertigo respectively, after adjusting for age, sex, income, geographic location, residential urbanization level, hypertension, coronary heart disease and diabetes. We did not find increased odds of sudden hearing loss for patients with PM/DM (adjusted OR = 0.889, 95% CI = 0.521–1.520).

**Table 3:** Covariate-adjusted odds of tinnitus, non-conductive hearing loss, vertigo, and sudden deafness among polymyositis/dermatomyositis versus comparison patients.

Variable	Odds Ratio (95% confidence interval, CIs) n = 9731			
	Tinnitus	Sudden deafness	Non-conductive hearing loss	Vertigo
Polymyositis/dermatomyositis (unadjusted)	1.321^c^ (1.140–1.531)	0.888 (0.520–1.515)	1.380^c^ (1.142–1.668)	1.351^c^ (1.157–1.577)
Polymyositis/dermatomyositis (adjusted*)	1.332^c^ (1.147–1.547)	0.889 (0.521–1.520)	1.399^c^ (1.154–1.696)	1.374^c^ (1.173–1.611)
Polymyositis (unadjusted)	1.433^c^ (1.174–1.751)	0.834 (0.385–1.806)	1.372^a^ (1.056–1.784)	1.589^c^ (1.296–1.949)
Polymyositis (adjusted*)	1.471^c^ (1.199–1.804)	0.853 (0.392–1.856)	1.506^b^ (1.151–1.970)	1.733^c^ (1.403–2.142)
Dermatomyositis (unadjusted)	1.212 (0.994–1.478)	0.940 (0.472–1.872)	1.377^a^ (1.076–1.762)	1.160 (0.938–1.434)
Dermatomyositis (adjusted*)	1.209 (0.988–1.480)	0.941 (0.470–1.884)	1.317^a^ (1.023–1.696)	1.125 (0.903–1.400)

*Adjusted for age, sex, monthly income, geographic location, urbanization level, hypertension, diabetes, and coronary heart disease.

^a^*p* < 0.05, ^b^ *p* < 0.01, ^c^ *p* < 0.001.

Table 3 further presents the associations separately for the subgroup with PM (i.e., excluding patients with PM from analysis). Patients with PM were more likely to have tinnitus (adjusted OR = 1.471, 95% CI = 1.199–1.804), non-conductive hearing loss (adjusted OR = 1.506, 95% CI = 1.151–1.970), and vertigo (adjusted OR = 1.733, 95% CI = 1.403–2.142) than comparison patients. After adjusting for the demographic variables as above. Limiting the analysis to DM patients and comparison patients, we found statistically significant associations of DM with non-conductive hearing loss (adjusted OR = 1.317, 95% CI = 1.023–1.696), but not with tinnitus (adjusted OR = 1.209, 95% CI = 0.988–1.480), sudden deafness (adjusted OR = 0.941, 95% CI = 0.470–1.884) or with vertigo (adjusted OR = 1.125, 95% CI = 0.903–1.400).

Table 4 analyzed the odds of tinnitus, non-conductive hearing loss, vertigo, and sudden deafness among polymyositis/dermatomyositis vs. comparison patients according to age group. Of the patients < 65 years old, we found statistically significant associations of PM/DM with tinnitus (adjusted OR = 1.290, 95% CI = 1.085–1.535) and non-conductive hearing loss (adjusted OR = 1.461, 95% CI = 1.149–1.857), but not with sudden deafness or with vertigo. Furthermore, we only found a statistically significant association of PM/DM with tinnitus (adjusted OR = 1.467, 95% CI = 1.084–1.987), but not with non-conductive hearing loss, sudden deafness, or with vertigo on patients ≥ 65 years old.

**Table 4:** Covariate-adjusted odds of tinnitus, non-conductive hearing loss, vertigo, and sudden deafness among polymyositis/dermatomyositis vs. comparison patients according to age.

Variable	Odds Ratio (95% confidence interval, CIs)			
	Tinnitus	Sudden deafness	Non-conductive hearing loss	Vertigo
Age < 65,* n* = 7806				
Polymyositis/dermatomyositis (unadjusted)	1.280^b^ (1.079–1.518)	1.066 (0.584–1.946)	1.450^b^ (1.142–1.840)	1.066 (0.584–1.946)
Polymyositis/dermatomyositis (adjusted*)	1.290^b^ (1.085–1.535)	1.069 (0.585–1.955)	1.461^b^ (1.149–1.857)	1.069 (0.585–1.955)
Polymyositis (unadjusted)	1.378^b^ (1.099–1.727)	1.000 (0.431–2.322)	1.493^a^ (1.087–2.050)	1.000 (0.431–2.322)
Polymyositis (adjusted*)	1.389^b^ (1.103–1.749)	1.016 (0.435–2.372)	1.506^a^ (1.093–2.077)	1.016 (0.435–2.372)
Dermatomyositis (unadjusted)	1.165 (0.921–1.475)	1.138 (0.518–2.497)	1.395^a^ (1.003–1.912)	1.138 (0.518–2.497)
Dermatomyositis (adjusted*)	1.178 (0.927–1.498)	1.125 (0.509–2.486)	1.399^a^ (1.009–1.939)	1.125 (0.509–2.486)
Age ≥ 65, *n* = 1925				
Polymyositis/dermatomyositis (unadjusted)	1.462^a^ (1.087–1.967)	0.512 (0.155–1.692)	1.289 (0.935–1.776)	0.512 (0.155–1.692)
Polymyositis/dermatomyositis (adjusted*)	1.467^a^ (1.084–1.987)	0.506 (0.152–1.685)	1.300 (0.939–1.800)	0.506 (0.152–1.685)
Polymyositis (unadjusted)	1.850^b^ (1.195–2.846)	0.476 (0.064–3.529)	1.443 (0.888–2.345)	0.476 (0.064–3.529)
Polymyositis (adjusted*)	1.812^b^ (1.153–2.846)	0.455 (0.060–3.443)	1.513 (0.918–2.493)	0.455 (0.060–3.443)
Dermatomyositis (unadjusted)	1.271 (0.877–1.840)	0.535 (0.127–2.259)	1.212 (0.818–1.798)	0.535 (0.127–2.259)
Dermatomyositis (adjusted*)	1.303 (0.890–1.908)	0.564 (0.131–2.424)	1.208 (0.808–1.805)	0.564 (0.131–2.424)

*Adjusted for age, sex, monthly income, geographic location, urbanization level, hypertension, diabetes, and coronary heart disease.

^a^*p* < 0.05, ^b^*p* < 0.01, ^c^*p* < 0.001.

## Discussion

Our study shows that patients with PM/DM have higher prevalence of tinnitus, non-conductive hearing loss, and vertigo. When patients with PM and DM were analyzed separately, we found that while patients with either PM or DM were more likely to have tinnitus and non-conductive hearing loss, the prevalence of vertigo was higher among PM patients relative to comparison patients but not among patients with DM. Our study suggests a substantial magnitude of association between PM/DM and inner ear disease which may be due to shared disease pathogenesis mechanisms. Our study finding may have some clinical value in enabling potentially early diagnosis and prevention of otolaryngological complications among patients with DM and PM.

As early as 1984, Veldman et al. mentioned polymyositis (PM) and dermatomyositis (DM) as autoimmune disorders which may affect the inner ear^16^. PM and DM are rare autoimmune inflammatory myopathies characterized by muscle weakness due to inflammation, and known to be associated with multisystem complications, including interstitial lung disease and cardiovascular disease^13,14,17–20^. Complications of the peripheral nervous system in PM/DM were first described as neuromyositis by Senator in 1893, and recently described in several case reports. Studies suggest an escalated frequency of cancer development among patients with DM and slightly increased frequency among those with PM^19,20^. Thus far there is little documentation of the clinical presentation and pathogenesis of peripheral neuropathy among patients being treated for PM or DM^19,21,22^. In 2017, Dhawan et al*.* reported a case study of systemic vasculitis occurring with dermatomyositis, hearing loss, neuropathy, and other multiorgan dysfunction^23^. This remains the only report of an association between DM and hearing loss. The authors did not explore likely pathogenic mechanisms in the report. Matsui et al. proposed that VEGF overproduction may drive the development of vasculitis among patients with DM complicated with peripheral neuropathy^24^. Vogelgesang et al.^25^ described the capillary endothelial ischemia found in nerve biopsy samples mimicked the pathologic findings of muscle tissue biopsy.

Some researchers have hypothesized that the formation of membrane attacking complexes brought about by deposition of C5b-9 around small blood vessels and capillaries in the endoneurium may cause complement-mediated damage, a mechanism that supports a common underlying pathogenic mechanism of both muscle and nerve injury in DM^25,26^. Other authors have reported the presence of immunoglobulin G (IgG) antibody and complement in endolymphatic fluid. Gutierrez et al. demonstrated that patients with endolymphatic hydrops had elevated levels of circulating immune complex, and proposed that endolymphatic hydrops may be caused by circulating immune complexes^27^. Our findings suggest that PM/DM may impact inner ear pathology by damaging the vestibulo-cochlear blood vessels and peripheral nerves partly based on the above mechanisms.

We found a higher prevalence of vertigo among patients with PM but not DM. There is little documentation comparing PM and DM on the severity of neurologic manifestations. McGarvey et al. reported differences in the anatomic distribution and severity of muscle weakness in DM and PM, specifically greater severity of proximal muscle group weakness in PM than in DM^28^. This finding is compatible with our findings suggesting more severe AIED complications among patients with PM^28^. Recent studies, using a clinical-serological approach, have characterized both DM and PM patients into more homogeneous subsets^29^. Further investigation of these subgroups may provide pointers to variations in disease progression which may explain our findings.

Patients with DM are often screened for nasopharyngeal lesions. Based on claims data from Taiwan’s National Health Insurance program, in 2009, Huang et al. reported that patients with DM were more likely to have nasopharyngeal carcinoma, lung cancer, and breast cancer. Patients with PM were shown to have greater risk of breast, uterine cervix, and lung cancers. Compared with the general population, DM conferred a tenfold higher risk for cancers, and among all cancers, a 66-fold higher risk of nasopharyngeal carcinoma and a 31-fold higher risk for lung cancer were particularly noteworthy^30^. A subsequent study showed that PM/DM is associated with an increased risk of NPC in South Asian countries^31^. Several hypotheses were proposed, including a possible role of Epstein-Barr virus (EBV), which is found in NPC and also a factor implicated in promoting the pathology that leads to PM/DM. In their study, most of the patients appear to have developed NPC concurrently myositis, the two diagnoses separate by less than 12 months, and further, successful treatment of NPC resulted in remission of myositis in 53.8% of patients^31^.

Our findings suggest that in addition to screening myositis patients for nasopharynx lesions, it may be useful to evaluate them for hearing and vestibular disorders. The optimal treatment of PM/DM patients complicated with hearing or vestibular disorders remains a question. At this point, glucocorticoid therapy remains the first line of treatment for hearing and balance disorders, and the only line of treatment for sudden hearing loss, Meniére's disease, immune-mediated hearing loss, and any vestibular dysfunction suspected of having an inflammatory etiology^6,32^. Immunosuppressive and immunomodulatory drugs are considered adjuvant or second-choice treatments, especially in cases complicated by active tuberculosis, diabetes, or hypertension, or failure of corticosteroid treatment or a need for very high dose to control the disease^6,7,32^. Many studies document the effectiveness of cyclophosphamide and azathioprine in improving hearing loss, however, marred by serious side effects. Newer tumor necrosis factor (TNF) inhibitors show good efficacy and fewer side effects.6 Infliximab seems to be the most promising biological remedy, enabling steroid tapering and improving auditory disease, with better results when administered in the early stages^6,9,33^. Our findings may suggest that given the higher likelihood of hearing and vestibular disorders among patients with PM/DM, more active use of other immunosuppressive therapy may add value in terms of stabilizing the pathology and preventing inner ear complications once the diagnosis of PM/DM has been made.

On another note, whether a PM/DM complicated with inner ear comorbidities represents an indicator of greater disease severity also needs further investigation. Variables associated with poor outcomes of PM/DM are documented, older age, pulmonary and esophageal involvement, and cancer^34^.

Another possibility is that the audiovestibular symptoms may not only be initial presentation of PM/DM, but may also serve as a disease severity or prognostic indicator. If audiovstibular symptoms are detected early in the course of PM/DM, it is possible that early pursuit of a more aggressive disease control approach may improve the likelihood of preventing irreversible damage to the inner ear and decrease severe PM/DM complications.

There are some study limitations. First, typically, studies based on health insurance claims are likely to have surveillance bias—patients with tinnitus or other hearing disorders and those with PM/DM have greater exposure to health services, particularly otolaryngology specialists for the purposes of nasopharyngeal cancer screening purposes. These patients would be more likely questioned about and complain about their related ear problems, resulting in greater likelihood of ear-related investigation. The study from Taiwan using health registry data and ICD codes highlights the risk of misclassifying myositis patients, including cases of Inclusion-Body Myositis and other myopathies, especially in outpatient settings. The lack of additional diagnostic criteria like serum enzyme levels or specialist consultations leads to inaccuracies, impacting both inpatient and severe cases. In Taiwan's healthcare system, accurate myositis diagnosis is critical, as it determines eligibility for a Catastrophic Illness Certificate, which exempts patients from medical copayments, and thus, we believe that the diagnosis of PM/DM carries significant weight in Taiwan's healthcare system.

Second, claims data have no data on the severity of the ear-related problems. Therefore, whether these patients with DM or PM actually have significant ear-related comorbidity cannot be determined form claims data. Third, we did not account for the medications used for the management of PM/DM. Typically used are azathioprine and methotrexate, which are reported to have adverse effects in the form of ear-related complications^35^. Finally, it is worth mentioning that within our investigation, 147 (17.0%) and 102 (13.4%) of patients diagnosed with dermatomyositis and polymyositis, respectively, had previously been diagnosed with a malignant condition. Given that malignancies and their treatments may also play a role in the emergence of hearing loss, tinnitus, and vertigo symptoms, certain biases could arise under these circumstances.

Large follow-up studies of clinical cohorts are needed to enhance our understanding of the etiopathogenesis of AIEDs and the pathological relationship between inner ear disorders and DM/PM. In the interim, our study suggests that increasing clinicians’ awareness of the likelihood of co-occurring PM/DM and vestibular disorders of autoimmune origin may be useful by keeping them alert to the possibility of underlying autoimmune processes driving both ear disease and PM/DM when patients report multiple and diverse symptoms. This could help early mitigation of the underlying processes and improve the quality of life of these patients.

In conclusion, patients with PM/DM show higher prevalence of tinnitus, non-conductive hearing loss, and vertigo. More research is needed to validate the finding and to investigate the causal mechanisms driving the association.

## Data Availability

Data from the National Health Insurance Research Database, now managed by the Health and Welfare Data Science Center (HWDC), can be obtained by interested researchers through a formal application process addressed to the HWDC, Department of Statistics, Ministry of Health and Welfare, Taiwan (https://dep.mohw.gov.tw/DOS/lp-2506-113.html 02/01/2022).
